# A Case-Based Review of Arthritis Robustus

**DOI:** 10.7759/cureus.50583

**Published:** 2023-12-15

**Authors:** Vijay Karthik Bhogaraju, Arnav Kalra, Divya Sindhuja Pathuri, Sukdev Manna, Venkatesh S Pai

**Affiliations:** 1 Internal Medicine/Clinical Immunology and Rheumatology, All India Institute of Medical Sciences, Rishikesh, Rishikesh, IND; 2 Internal Medicine, All India Institute of Medical Sciences, Rishikesh, Rishikesh, IND; 3 Ophthalmology, All India Institute of Medical Sciences, Rishikesh, Rishikesh, IND

**Keywords:** peripheral arthritis, person-centered care, clinical rheumatology, rheumatoid arthritis, arthritis robustus

## Abstract

Rheumatoid arthritis (RA) commonly presents as a chronic additive symmetric inflammatory polyarthritis involving the small and large joints. Rarely do patients present with few or no clinical symptoms, despite apparent signs of inflammation. This condition, known as arthritis robustus, typically occurs in elderly males who are manual laborers with an active lifestyle. It is essential to diagnose arthritis robustus and start treatment promptly to avoid the development of deformities and other complications in the future.

## Introduction

Arthritis robustus (rheumatoid robustus) commonly occurs in men over the age of 50, particularly those who are physically active and involved in manual labor. They do not complain of pain, stiffness, disability, or distress, though clinical signs of inflammation, deformity, and radiological erosions are present. Synovial proliferation, subcutaneous nodules, periarticular erosions, and subchondral cysts are common, while periarticular osteopenia is rare compared to classical rheumatoid arthritis (RA) [1].

We detail a unique case of arthritis robustus with tenosynovitis, review published literature, discuss potential reasons for the unique presentation, and explore the clinical and therapeutic implications.

## Case presentation

**Figure 1:**
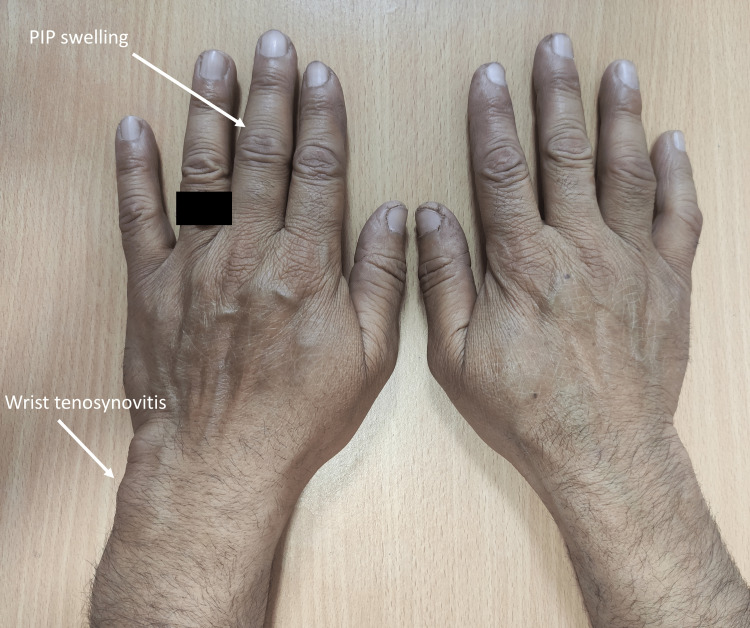
Both wrists of the patient Note the left 3rd PIP swelling and left wrist tenosynovitis. PIP: proximal interphalangeal

A 45-year-old male was referred to the rheumatology clinic for complaints of swelling in both wrists for a six-month duration. The swelling persisted without progressing, and there was no associated joint pain. However, the patient experienced 30 minutes of early morning stiffness in the wrists and small joints of the hands. There was no associated history of fever, back pain, skin lesions, diarrhea, or urethral discharge. He worked as a milkman and was a former smoker who smoked 20 hand-rolled cigarettes (bidi) daily for ~ 10 years. Past medical and surgical history was unremarkable. On examination, swelling with fluctuation was present on both wrists and the left third proximal interphalangeal (PIP) joint, associated with warmth but without tenderness. A firm, non-tender swelling of size 1 x 1 cm was present over the left distal ulna (Figure 1). Radiographs of the bilateral wrist confirmed joint space narrowing with erosions but without significant periarticular osteopenia suggesting long-standing inflammatory disease.

Investigations were remarkable for high rheumatoid factor (RF >90 IU/mL), anti-cyclic citrullinated peptide (CCP) antibodies (>80 IU/mL), and serum C-reactive protein (CRP) (8.2 mg/L) values (Table 1). Musculoskeletal ultrasonography revealed tenosynovitis of the left extensor carpi ulnaris tendon and cortical irregularity in the phalanges. The patient fulfilled the 2010 ACR/EULAR classification criteria with a DAS28-CRP score of 3.25, suggesting moderate disease activity.

**Table 1:** Investigations at presentation ESR: erythrocyte sedimentation rate; TLC: total leucocyte count; DLC: differential leucocyte count; CRP: C-reactive protein; ALT: alanine transaminase; AST: aspartate transferase; ALP: alkaline phosphatase; GGT: gamma-glutamyl transferase; CCP: cyclic citrullinated peptide; HCV: hepatitis C virus

Test	Value	Test	Value
Hb(g/dL)	12.7	S. Uric acid	6.7 mg/dL
TLC per mm^3^	10,590/mm^3^	ESR	14 mm in 1st hour
DLC (%)	N 69%, L 24%, M 6%, E 1%	S. CRP	8.2 mg/L
Platelet count/mm^3^	3,35,000	Rheumatoid factor (RF)	>90 IU/mL (<14 )
S. Bilirubin (T/D) mg/dL	0.30/0.18	Anti-CCP	>80 IU/mL (<5 )
ALT/AST(IU/L)	22.1/ 26.6	Anti-HIV-1 & -2	Non-reactive
ALP/GGT (IU/L)	337.3/ 60.4	HBsAg	Non-reactive
S. Total protein/ S. Albumin g/dL	8.02/4.04	Anti-HCV	Non-reactive

The patient was counseled and educated regarding this condition and was initiated on methotrexate, bridge therapy with low-dose prednisolone, and nonsteroidal anti-inflammatory drugs (NSAIDs) for RA. He was provided with physical and occupational therapy. The dose of prednisolone was tapered on follow-up and discontinued over a period of two months. The patient did not report a need for analgesics on follow-up visits. At the third-month follow-up visit, the patient’s swollen joint count was reduced to zero with disease activity measure DAS28-CRP 1.64 (suggestive of remission).

## Discussion

Arthritis robustus primarily affects elderly males actively involved in physical labor. Despite the presence of an inflammatory joint disease that aligns with this patient’s clinical profile, the level of pain and stiffness is minimal. Also, a history of smoking was a risk factor for the development of RA [2]. Our patient is a milkman who uses small hand joints during milking. Continuous use of these joints would affect the perception of pain and stiffness, reducing awareness of symptoms.

De Haas et al. described nine male patients with classical RA who had subcutaneous nodules as well as high titers of seropositivity of both RF/anti-CCP [3]. Despite these characteristics, they were “robust, healthy, and working normally.” The duration of arthritis robustus and joint involvement showed no significant difference compared to controls, who were males experiencing both active disease and remission. However, all of them were involved in strenuous physical work, had a mesomorphic body structure, and demonstrated a tendency toward independence during psychological interviews. These authors interrogated if these clinical features could be explained by the “soft-hearted” treatment of some patients. It was reported, however, that “RA, typus robustus” men needed fewer analgesics and less physiotherapy.

Chopra and Chib reported a case of arthritis robustus, masquerading as gout [4]. The term “arthritis robustus variant of RA” was applied to categorize four out of 20 young servicemen experiencing chronic inflammatory polyarthritides. It must be noted that these were physically active youthful men, and the prevalence of “robustness” was four out of 15 RA patients [5]. More recently, Prasad et al. reported a 58-year-old telephone wireman with an active lifestyle who had clinically evident arthritis without arthralgia, diagnosed as arthritis robustus only when he presented with myocardial infarction [6]. Thompson and Carr reported 10 patients, out of a randomly selected list of 100 RA patients, who did not complain of pain despite having clinical and biochemical evidence of inflammation [7]. Jones takes this discussion forward to propose the role of psychosocial factors in pain perception and management [8]. Earlier, authors have highlighted the correlation between pain threshold and analgesic usage in men with RA, as opposed to those with ankylosing spondylitis, where pain threshold does not influence analgesic requirement [9].

A large study looked into the discordance of self-reported symptoms with objective disease activity scores/inflammatory markers. This study looked at three cohorts, namely (1) the Early Rheumatoid Arthritis Network (ERAN), (2) the British Society for Rheumatology Biologics Registry (BSRBR), starting therapy with tumor necrosis factor (TNF) inhibitors, as well as (3) those on non-biologic medications. A subset of patients with discordantly better patient-reported outcomes (PRO) compared to inflammation was identified, including 11% in the ERAN cohort, 23% in the BSRBR cohort of TNF inhibitors, and 10% in (BSBR) non-biologic medications. This suggested that non-inflammatory factors may influence the interpretation of inflammation/pain, acknowledging the presence of the typus robustus RA phenotype in this subset. It is worth noting that the authors place a greater emphasis on the needs of another subset that reported discordantly worse (in contrast to arthritis robustus) PRO compared to inflammation (12%, 40%, and 21% in the three cohorts) [10].

The pathogenesis of RA is multifactorial, with putative inflammatory and non-inflammatory pathways contributing to its clinical phenotype. Individuals with arthritis robustus may have lower interleukin-6-mediated dysfunction, which changes their pain perception [11] (Table 2). Healthcare-seeking behavior, too, may influence the presentation of arthritis. Patients with less access to rheumatology care may tend to downplay their concerns. The same applies to individuals who cannot afford quality care. One differential diagnosis of arthritis robustus is leprosy, which can present with deforming arthritis and lack of symptoms due to sensory impairment [12]. It may be possible that arthritis associated with sensory neuropathy, e.g., syphilis, diabetes, and acromegaly, can mimic arthritis robustus. Endemic diseases, such as nutritional and toxic neuropathy, may impact the perception of normal vs. abnormal, delaying their recognition of symptoms and signs [13-15]. Thus, arthritis robustus can be explained by the biopsychosocial model of health [16] (Table 2).

**Table 2:** Potential explanations for arthritis robustus based upon the biopsychosocial model of health

Biomedical
Related to rheumatoid arthritis: lack of non-inflammatory pain pathways
Associated sensory neuropathy, e.g., leprosy, syphilis, diabetes, nutritional deficiency, alcoholism
Higher pain threshold
Psychosocial
Independent personality
Delayed healthcare-seeking behavior
Acceptance of joint-related symptoms due to the ubiquitous prevalence

Arthritis robustus has important clinical implications. Delayed recognition of the disease may lead to the progression of the disease and the occurrence of complications, including deformities. It may also result in delayed identification of comorbid conditions, such as autoimmune disorders, osteopenia, and sarcopenia, potentially leading to further morbidity. An early diagnosis allows the timely institution of disease-modifying anti-rheumatic drugs (DMARDs), and appropriate lifestyle changes, which may optimize long-term health. The construct of arthritis robustus also allows healthcare professionals to individualize therapy in a person-centered manner [16]. Arthritis robustus patients, for treatment adherence, require motivation, encouraging a proactive approach to managing their condition and controlling disease activity.

## Conclusions

The current case of arthritis robustus has associated tenosynovitis, which is rare. Apart from highlighting the existence of this syndrome, this discussion also underscores the need to spread awareness about this variant of RA while understanding its clinical presentation, differential diagnosis, and management.
